# EuroSCORE II is a poor predictor of peroperative outcomes in octogenarians after cardiac surgery

**DOI:** 10.1186/1749-8090-8-S1-O125

**Published:** 2013-09-11

**Authors:** A Rösler, M Sales, J Frota Filho, J Fraportti, G Constantin, E Lúcio, P Leães, M Pontes, F Lucchese

**Affiliations:** 1 Cardiovascular Surgery, Hospital São Francisco, Porto Alegre, Brazil

## Background

Octogenarian patients undergoing cardiac surgery have higher morbidity and mortality rates. Risk prediction tools are not well validated in this age group. Our goal was to evaluate predictive ability of EuroSCORE II (ES II) for in-hospital outcomes in octogenarian patients after cardiac surgery.

## Methods

Consecutive octogenarians operated on in our hospital from 2006 to 2012, excluding surgery of aorta. We evaluated demographics, clinical and operative variables, ES II, and in-hospital outcomes. Predictive ability of ES II was evaluated for clinical performance (observed/expected mortality ratio), calibration (Hosmer-Lemeshow test) and accuracy (area under ROC curve – AUC).

## Results

We included 192 octogenarians (83±3y, 53% male, EF 61±13%. ES I=10,1 [7,9-17,2] , ES II=3,6 [2,6-5,5]. Surgeries included CABG (35,9%), Valve (41,1%), CABG + valve (44, 22,9%). The observed in-hospital outcomes were: early reoperation (5,2%), MACCE (12,5%), in-hospital mortality (12,0%). ES II was higher in CABG + valve group compared to isolated CABG and valve groups (6,1±4,8% vs 4,2±3,6% e 4,5±3,4%, p=0,028). Corresponding observed mortality rates were 20,5%, 11,6%, and 7,6% (p=0,108). Clinical performance of ES II was poor. O/E ratio was: entire cohort (2,55), CABG (2,76), Valve (1,69), CABG + valve (3,36), p<0,05 for all comparisons. Patients were stratified according to ES I-defined risk (low risk < 5, medium risk 5 - 9,9, high risk 10 – 14,9, very high risk > 15). Calibration of ES II in these strata were 0; 2,88; 2,50; e 2,6, respectively (Hosmer-Lemeshow p<0,05). Accuracy of ES II was a bit higher than ES I, but both scores showed poor discriminatory power (AUC ROC 0,686 e 0,647, respectively).

## Conclusions

In octogenarians, ES II underestimated surgical risk, showed low accuracy and poor calibration in all strata of risk. New specific validated risk-prediction tools are needed in order to perform accurate risk stratification in this age group.

